# Circulating miRNAs as a Predictive Biomarker of the Progression from Prediabetes to Diabetes: Outcomes of a 5-Year Prospective Observational Study

**DOI:** 10.3390/jcm9072184

**Published:** 2020-07-10

**Authors:** Iwona Sidorkiewicz, Magdalena Niemira, Katarzyna Maliszewska, Anna Erol, Agnieszka Bielska, Anna Szalkowska, Edyta Adamska-Patruno, Lukasz Szczerbinski, Maria Gorska, Adam Kretowski

**Affiliations:** 1 Clinical Research Centre, Medical University of Bialystok, 15-276 Bialystok, Poland; anna.erol@umb.edu.pl (A.E.); agnieszka.bielska@umb.edu.pl (A.B.); anna.szalkowska@umb.edu.pl (A.S.); edyta.adamska@umb.edu.pl (E.A.-P.); lukasz.szczerbinski@umb.edu.pl (L.S.); adamkretowski@wp.pl (A.K.); 2 Department of Endocrinology, Diabetology and Internal Medicine, Medical University of Bialystok, 15-276 Bialystok, Poland; maliszewska.k@gmail.com (K.M.); mgorska25@wp.pl (M.G.)

**Keywords:** diabetes, prediabetes, miRNA, serum profiling, biomarker

## Abstract

Due to a global increase in the prevalence of type 2 diabetes mellitus (T2DM), there is an urgent need for early identification of prediabetes, as these people have the highest risk of developing diabetes. Circulating miRNAs have shown potential as progression biomarkers in other diseases. This study aimed to conduct a baseline comparison of serum-circulating miRNAs in prediabetic individuals, with the distinction between those who later progressed to T2DM and those who did not. The expression levels of 798 miRNAs using NanoString technology were examined. Spearman correlation, receiver operating characteristic (ROC) curve analysis, and logistic regression modeling were performed. Gene ontology (GO) and canonical pathway analysis were used to explore the biological functions of the miRNA target genes. The study revealed that three miRNAs were upregulated in the serum samples of patients who later progressed to T2DM. Pathway analysis showed that the miRNA target genes were mainly significantly enriched in neuronal NO synthase (nNOS) signaling in neurons, amyloid processing, and hepatic cholestasis. ROC analysis demonstrated that miR-491-5p, miR-1307-3p, and miR-298 can be introduced as a diagnostic tool for the prediction of T2DM (area under the curve (AUC) = 94.0%, 88.0%, and 84.0%, respectively). Validation by real-time quantitative polymerase chain reaction (qRT-PCR) confirmed our findings. The results suggest that circulating miRNAs can potentially be used as predictive biomarkers of T2DM in prediabetic patients.

## 1. Introduction

Diabetes mellitus is a chronic metabolic disease affecting more than 450 million people worldwide. It is caused by defects in insulin production, secretion, and signaling [1]. There are two principal types of diabetes—type 1 diabetes mellitus (T1DM) and type 2 diabetes mellitus (T2DM). T2DM is a complex polygenic disease and involves interplay between genetic, epigenetic, and environmental factors. The disease is characterized by increased blood glucose levels (hyperglycemia) as a result of impaired insulin secretion due to pancreatic beta (β)-cell dysfunction and insulin resistance [2]. Before developing T2DM, individuals undergo an intermediate state termed prediabetes, characterized by abnormal glucose homeostasis, which is lower than the diabetes threshold [3,4]. What should be emphasized is that not all prediabetic patients progress to diabetes; however, many of them have a higher risk of developing diabetes in the future [5]. The clinical phenomenon of a complex metabolic disease like T2DM is delayed by years, thereby restricting its timely diagnosis. Therefore, not only can individual risk factors for T2DM (e.g., obesity, first degree relative with diabetes, and hypertension) be used to define populations at-risk for diabetes, but also, a combination of risk factors, named metabolic syndrome (MetS). MetS is a cluster of several characteristics, including obesity (especially abdominal adiposity), glucose intolerance, insulin resistance, dyslipidemia, microalbuminuria, hypertension, nonalcoholic fatty liver disease (NAFLD), polycystic ovarian syndrome, the proinflammatory state, and oxidative stress, resulting in an increased risk of T2DM [6,7,8,9]. However, their predictive value is poorer than that of prediabetes [10].

Prolonged hyperglycemia, often asymptomatic, leads to microvascular, macrovascular, and neurological complications, which are the major symptoms of T2DM that decrease life expectancy in individuals [11,12,13]. Classical diagnostic markers for T2DM are generally detected late, after the metabolic imbalance has occurred [14]. An increased incidence of prediabetes and T2DM has raised an imperative need for early detection and for prediction of the progression from prediabetes to T2DM. A specific biomarker could be useful in the recognition of susceptible individuals. Therefore, delaying the symptoms of diabetes could be achieved by implementing preventive interventions and effective treatment as early as possible [15,16,17].

miRNAs belong to a class of highly conserved, sequence-specific, single-stranded, endogenous small non-coding RNAs (18–25 nucleotides in length). They have been shown to regulate eukaryotic gene expression by binding to the 3’ end of the target mRNAs to induce their destabilization, degradation, and/or inhibit translation [18]. miRNAs can regulate multiple genes and are involved in various important signaling pathways. Several miRNAs are known to be involved in the pathogenesis of T2DM, and their role in diabetes complications has been proven [19]. Kamalden et al. reported that miR-15 plays an important role in insulin secretion in pancreatic β-cells. Additionally, miR-15 has been shown to be produced in pancreatic β-cells and to enter the bloodstream, and, therefore, to contribute to retinal injury during the development of T2DM [20]. The expression of miR-124a is upregulated in human type 2 diabetic pancreatic islets, suggesting that miR-124a negatively regulates glucose-induced insulin secretion [21]. The literature data show that miR-375 regulates glucose homeostasis, insulin secretion, and the development, maintenance, and survival of pancreatic β-cells [22]. The majority of miRNAs have been found intracellularly; miRNAs are actively and selectively released from cells in response to stress or injury, thus can function as signaling molecules in health and disease [18]. The fact that tissue-specific miRNAs may enter the circulation system, including blood and other body fluids, has opened up the possibility of using circulating miRNAs as non-invasive predictors of disease progression [23]. Jimenez-Lucena et al. found that circulating miRNA levels combined with glycated hemoglobin A1c (HbA1c) could be potentially used for predicting T2DM development [24]. Additionally, miRNAs have also been reported to be related to prediabetes. For example, miR-126 and miR-15a levels have been shown to be significantly lower in prediabetes and T2DM [25,26]. However, the study by Kong et al. reported that seven diabetes-related serum microRNAs (miR-9, miR-29a, miR-30d, miR-34a, miR-124a, miR-146a, and miR-375), which play a key role in β-cell function, are not able to discriminate prediabetes patients from T2DM-susceptible individuals with normal glucose tolerance [27]. Interestingly, many of these miRNAs have also been identified as biomarkers for other metabolic diseases [28,29]. For instance, miR-15a-5p and miR-17-5p have been determined as predictive biomarkers of MetS [30]. Lin et al. showed an association between urinary miR-29a-3p levels and MetS [31]. Additionally, miR-126 has been demonstrated to be a useful marker of metabolic dysfunction in children with MetS traits [32]. The study by Al-Rawaf highlighted that regulation of the expression of distinct miRNAs is associated with adipokines and other related MetS metabolic factors [33]. Elevated miR-122 expression in young adults with obesity and insulin resistance has also been demonstrated [34]. Further studies have shown the role of several miRNAs as attractive potential biomarkers for treating obesity and associated risk factor diseases [35,36].

The broad goal of the current study was to identify circulating miRNAs that are associated with T2DM progression in prediabetes patients. Since the frequently used laboratory tests for risk stratification of prediabetes individuals are limited, there is an urgent need to identify a non-invasive biomarker that could be used to evaluate the risk of developing T2DM before clinical symptoms occur [37]. This would allow timely intervention, e.g., by lifestyle modification, to slow down the process of β-cell failure and to delay the onset of diabetes.

## 2. Materials and Methods

### 2.1. Study Subjects

All subjects were part of the 1000PLUS cohort gathered between 2009 and 2012 by the Department of Endocrinology, Diabetology and Internal Medicine, Medical University of Bialystok, Poland. The 1000PLUS cohort recruited 1151 Polish, Caucasian volunteers (583 women and 568 men), aged 18–71 (mean age of 44 years) with a known history of dysglycemia. Ethical approval for the study was obtained originally from the local Ethics Committee at the Medical University of Bialystok, Poland (R-I-002/290/2008/2009 and R-I-002/35/200). Before the study, all patients participating read and signed forms of informed consent. Five years after the first examination, 399 subjects from the 1000PLUS cohort underwent a follow-up. For this work, 42 prediabetes individuals were selected from the 1000PLUS study population who, at baseline, had not been clinically diagnosed with T2DM. A total of 24 subjects developed T2DM during the 5-year observation. The remaining group of subjects (*n* = 18) did not develop the disease. The presence of diabetes based on the dysglycemia diagnostic criteria of the Diabetes Poland was excluded at the beginning of the study and was confirmed or excluded after 5 years using glucose concentration measurements during an oral glucose tolerance test (OGTT) at 0 and 120 min [38]. The glucose homeostasis of the control group maintained as stable over the follow-up period. Patients with any infection, cardiovascular disease, recent surgery, or serious somatic disease were excluded. Patients with type 1 diabetes or latent autoimmune diabetes of adults were excluded. The study groups were homogeneous and did not differ regarding age and body mass index (BMI) status. The use of medication was not accepted at the time of the study.

### 2.2. Sample Collection and Measurement

The patients had fasted overnight and were asked to avoid intensive physical activity the day before the tests were conducted. Blood samples were collected on two visits (i.e., visit 1 and visit 2 (5 years later)) from all participants from whole-blood venipuncture into Sarstedt s-monovette serum-gel 7.5 mL tubes (Sarstedt, Mawson Lakes, SA, Australia). The tubes were processed within 2 h of collection and the serum was centrifuged. The OGTTs commenced with baseline blood collection (0 min), followed by oral consumption of a solution of 75 g of glucose dissolved in 300 mL of room-temperature water. Subsequent blood collections were taken at 120 min following glucose administration. Then, the blood samples were centrifuged for 10 min at 2000× *g* after complete blood coagulation, and the serum obtained was used for the following analysis. The samples were transferred to DNase- and RNase-free tubes (1.5 mL; Eppendorf, Hamburg, Germany). The cell-free serum samples were stored at –80 °C until being assayed. The hemolysis in all samples was tested by measurement of oxyhemoglobin absorbance at 414 nm, and hemolyzed samples were excluded.

Anthropometric measurements, including weight and BMI, were measured on visits 1 and 2 by standardized procedures. Biochemical measurements (i.e., plasma glucose, serum triglycerides (TG), total cholesterol, high-density lipoprotein (HDL), and low-density lipoprotein (LDL) concentrations) were performed by the colorimetric method with Cobas c111 (Roche Diagnostics, Basel, Switzerland). Insulin concentration was measured in the serum using an immunoradiometric assay (IRMA) kit (DIAsource ImmunoAssays SA, Belgium). The homeostatic model assessment for insulin resistance (HOMA-IR = fasting insulin (μU/mL) × fasting glucose (mmol/L)/22.5) and for beta (β) cell function (HOMA-β = (20 × fasting insulin (μU/mL))/(fasting glucose (mmol/L)) – 3.5) were calculated [39]. The concentration of glycated hemoglobin A1c (HbA1c) was measured by the high-performance liquid chromatography (HPLC) method (Bio-Rad VARIANT, Bio-Rad Laboratories, Hercules, USA).

### 2.3. Detection of the miRNA Profiles

miRNAs were isolated from the serum samples collected at baseline (on visit 1) using a miRNeasy Serum/Plasma Kit (Qiagen, Hilden, Germany) according to the manufacturer’s instructions. The RNA concentration was assessed by Qubit (Invitrogen, Carlsbad, USA). The samples were prepared for nCounter miRNA expression profiling according to the manufacturer’s recommendations (NanoString Technologies, USA). Briefly, 3 ng miRNA samples were prepared by ligating a specific miR-tag onto the 3′ end of each mature miRNA, followed by an overnight hybridization (65 °C) to nCounter Reporter and Capture probes. Subsequently, the samples were placed into the nCounter Prep Station for automated sample purification and subsequent reporter capture. Each sample was scanned on the nCounter Digital Analyzer for data collection. The NanoString data were deposited in the Gene Expression Omnibus (GEO) database (accession number: GSE148961).

### 2.4. Validation of the NanoString Results by Real-Time Quantitative Polymerase Chain Reaction (qRT-PCR)

For the validation experiments, the serum miRNA samples were reverse-transcribed using a miRCURY LNA RT Kit (Qiagen, Hilden, Germany), according to the manufacturer’s protocol, on a Proflex thermal cycler (Thermo Fisher Scientific, Waltham, USA). Then, the candidate miRNAs were quantified by qRT-PCR using specific primers and a miRCURY LNA SYBR Green PCR Kit (Qiagen, Germany). The samples were run on the qPCR plates in duplicate on the LightCycler 480 Real-Time PCR System (Roche, Switzerland). miR-103a-3p and miR-16-5p were used as endogenous control genes. The stability of the reference miRNAs was calculated by the NormFinder algorithm. Relative miRNAs expression was calculated using an efficiency corrected calculation model, based on multiple samples and multiple reference genes [40].

### 2.5. Data Analysis

Statistical analyses were performed with the R software package (version 3.3.2) and GraphPad PRISM (version 8.4.0). Preliminary statistical analysis (Shapiro–Wilk test) revealed that the studied parameters did not follow a normal distribution. Consequently, nonparametric tests were used for statistical analysis between groups. All data are presented as median and range. nSolver 4.0 Analysis software (NanoString) was used for data analysis, including normalization using the average geometric mean of the top 100 probes detected. The *p*-values were adjusted using the False Discovery Rate (FDR) correction for multiple comparisons, which was limited to 0.05. The threshold value for significance used to define upregulation or downregulation of miRNAs was a fold change >1.5. The Mann–Whitney U test was used to examine the statistical difference in the clinical parameters between patient samples. Spearman correlation was performed to measure the association between the clinical parameters and the miRNAs (package: corrplot Taiyun Wei and Viliam Simko (2017); R package “corrplot”: Visualization of a Correlation Matrix (Version 0.84); available from https://github.com/taiyun/corrplot). The ROC curves for all the differentially expressed miRNA were constructed, and the area under the ROC curves (AUCs) was calculated using package pROC [41]. Four logistic regression models out of a combination of the top three best performing miRNAs were prepared using leave-one-out-cross validation (LOOCV) with the help of package caret (Max Kuhn (2020); caret: Classification and Regression Training; R package version 6.0-86; https://CRAN.R-project.org/package=caret).

2.6. miRNA Target Prediction and Functional Annotation of the Selected miRNA Targets

To examine the functions of the identified miRNAs, miRNA target prediction was performed using Ingenuity Pathway Analysis (IPA) (QIAGEN Inc.). Gene ontology analysis (GO) and functional annotation clustering were carried out using the Gene Ontology enrichment analysis and visualization tool (GOrilla; http://cbl-gorilla.cs.technion.ac.il/), DAVID (Gene Ontology and KEGG (Kyoto Encyclopedia of Genes and Genomes) Enrichment Analysis; https://david.ncifcrf.gov/), g: Profiler (https://biit.cs.ut.ee/gprofiler/gos), and Metascape (https://metascape.org) online databases. GO analysis allows associating the given gene list with specific functional annotations, which are further divided into the functional clusters listed according to an enrichment *p*-value [42]. To identify highly connected hub genes in the protein–protein network (PPI), the CytoHubba plugin based on Cytoscape version 3.7.2 (http://cytoscape.org/) was used.

## 3. Results

### 3.1. Characteristics of the Patients

In the presented work, based on the 1000PLUS population study, 42 prediabetes patients—who, at baseline, had not been clinically diagnosed with T2DM—were included. Table 1 presents the median and the range of the anthropometric measurements and the biochemical parameters. At baseline, the groups did not differ significantly in terms of body mass index (BMI), weight, fasting glucose (FG), glucose concentration at 120′ during OGTT, fasting insulin, glycated hemoglobin (HbA1c), LDL, HDL, and total cholesterol, triglycerides, HOMA- β, and HOMA-IR. Taking into account the follow-up (visit 2), which was 5 years after visit 1, higher fasting glucose, glucose at 120′ during OGTT, and HbA1c concentrations were observed in the T2DM group compared to control the non-T2DM group. Other parameters, such as lipid profile, insulin, and HOMA indexes, showed no differences between the studied groups. 

### 3.2. Baseline Concentrations of Circulating miRNAs

The serum profiling of 798 miRNAs was performed using the NanoString Technology platform, and three of them were identified as differentially expressed between the studied groups (Table 2). Precisely, all of them were upregulated in the serum samples of patients who developed T2DM after 5 years compared to non-T2DM patients.

### 3.3. Canonical Pathway Analysis

**Figure 1:**
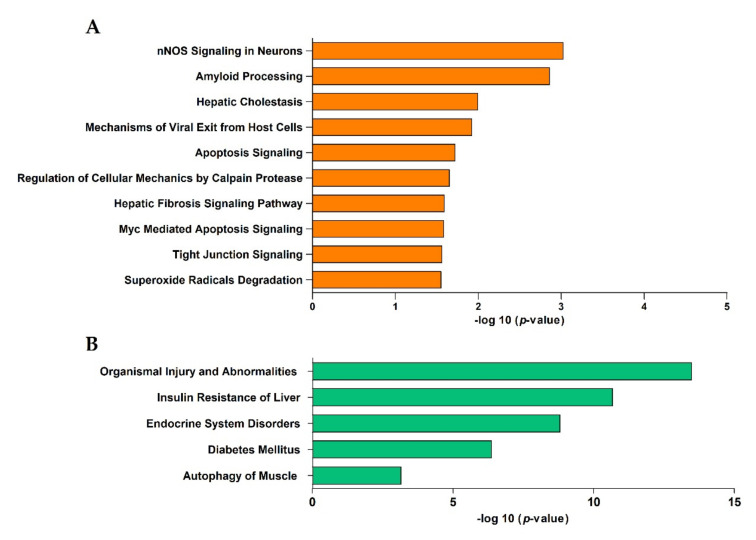
(**A**) The 10 most representative altered canonical pathways targeted by the differentially expressed (DE) miRNA target genes associated with the risk of T2DM development, identified using Ingenuity Pathway Analysis (IPA) (FDR < 0.05). (**B**) Top 5 diseases associated with the DE miRNA target genes (IPA analysis).

The prediction of the target genes of the three differentially expressed (DE) miRNAs was performed using Ingenuity Pathway Analysis (IPA), a software application for the analysis, integration, and interpretation of data from high-throughput experiments. The results indicate 822 putative target genes for the miRNAs. The ingenuity core analysis identified 20 altered canonical pathways at the baseline in the sera of patients who developed T2DM. The top ten canonical pathways associated with the DE miRNA target genes are listed in Figure 1A. Neuronal NO synthase (nNOS) signaling in neurons, amyloid processing, and hepatic cholestasis pathways were the most significantly changed. Using the IPA algorithms, the known diseases associated with the target genes were also identified. Interestingly, organismal injury and abnormalities, insulin resistance in the liver, endocrine system disorders, diabetes mellitus, and autophagy of muscles were the diseases most significantly linked to the DE miRNA target genes (Figure 1B).

### 3.4. Functional Enrichment Analysis

**Figure 2:**
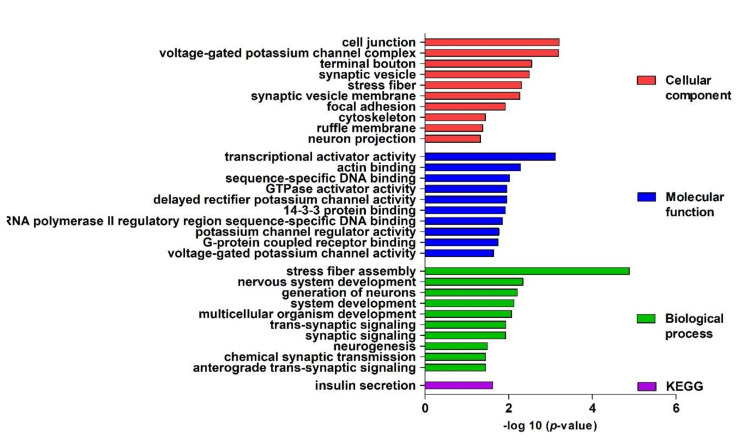
Gene ontology (GO) enrichment analysis. Top 10 significantly enriched GO (−log10 (*p*-value)) terms of the target genes in the cellular components, molecular function, and biological processes. KEGG, Kyoto Encyclopedia of Genes and Genomes.

Gene ontology was used for identifying and visualizing the appropriate biological pathways and processes associated with the target genes. The analysis of the T2DM network GO biological process terms revealed a predominant role of stress fiber assembly, nervous system development, generation of neurons, system development, multicellular organism development, trans-synaptic signaling, synaptic signaling, neurogenesis, chemical synaptic transmission, and anterograde trans-synaptic signaling. Regarding the molecular function terms, these genes are mainly enriched in transcriptional activator activity (RNA polymerase II core promoter proximal region sequence-specific binding), actin binding, sequence-specific DNA binding, GTPase activator activity, delayed rectifier potassium channel activity, 14-3-3 protein binding, G-protein coupled receptor binding, and voltage-gated potassium channel activity. The cellular component analysis identified primarily those involved in cell junctions, voltage-gated potassium channel complexes, terminal boutons, synaptic vesicles, stress fibers, synaptic vesicle membranes, focal adhesions, cytoskeletons, ruffle membranes, and neuron projections. Additionally, the KEGG pathway enrichment analysis confirmed that insulin secretion is involved in the development of T2DM in prediabetes patients (Figure 2).

### 3.5. Hub Gene Identification

**Figure 3:**
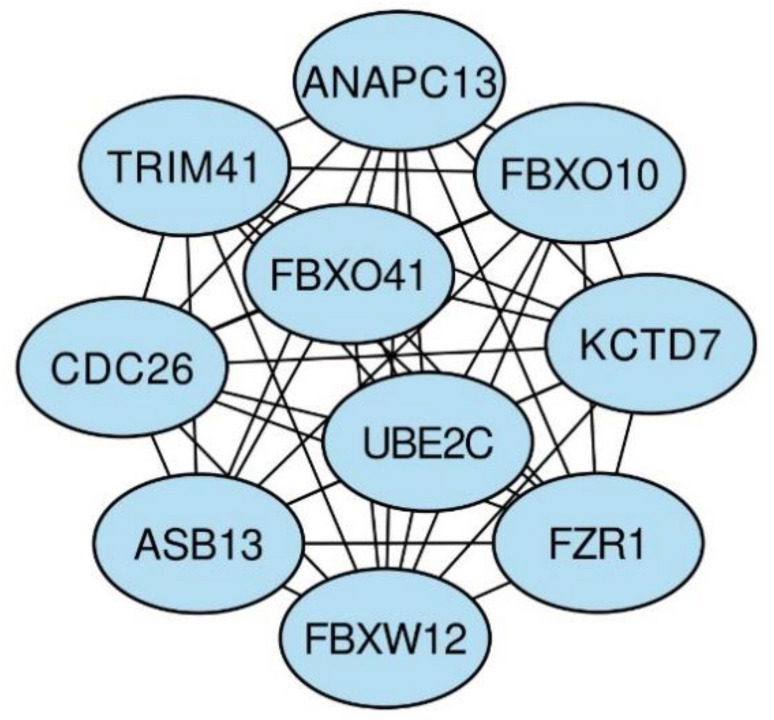
The networks of the top 10 hub genes.

Cytoscape software was used to visualize the construction of the PPI network of the differentially expressed (DE) miRNA target genes. Then, the Maximal Clique Centrality (MCC) algorithm in the cytoHubba plugin was used to filter the top 10 hub genes in the PPI network [43]. F-box proteins (*FBXO41*, *FBXO10*, and *FBXW12)*, *CDC26*, *TRIM41*, *KCTD7*, *ANAPC13*, *ASB13*, *UBEC2*, and *FZR1* were the top 10 *(*Figure 3).

### 3.6. Relationship between miRNA Serum Levels and Anthropometric and Biochemical Measurements

**Figure 4:**
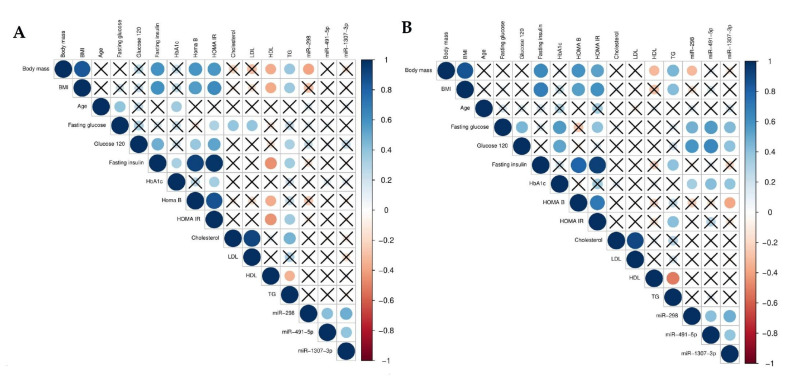
Graphical Spearman correlation matrix of the DE miRNAs (only baseline) and the other measurements on (**A**) visit 1 and (**B**) visit 2. The Spearman correlation r values were determined using R software.

A Spearman’s rank-order regression was performed to study the relationship between the miRNAs and both the baseline (visit 1) and the follow-up (visit 2) parameters. A negative correlation was observed between baseline body mass and HDL (r = −0.36, *p* < 0.05) and TG (r = −0.37, *p* < 0.05). Additionally, a positive correlation between BMI and fasting insulin concentration (r = 0.62; *p* < 0.001), HOMA-β (r = 0.56, *p* < 0.001), and HOMA-IR (r = 0.63, *p* < 0.001). Additionally, a correlation was demonstrated between glucose concentrations at 120′ during the OGTT and insulin concentration (r = 0.49, *p* < 0.001), HOMA-β (r = 0.37, *p* < 0.05), and HOMA-IR (r = 0.52, *p* < 0.01). Baseline body mass also correlated with HOMA-β (r = 0.59, *p* < 0.001) and HOMA-IR (r = 0.58, *p* < 0.001). Negative correlations between HDL cholesterol and fasting insulin concentration, HOMA-β, and HOMA-IR were observed (r = –0.46, r = –0.37, and r = –0.44, respectively; *p* < 0.01). Analysis revealed a moderate _negative_ correlation between miR-298 and body mass (r = –0.39, *p* > 0.05). However, a strong positive association was observed between the two DE miRNAs (miR-491-5p and miR-1307-3p; *p* < 0.01) (Figure 4A).

Taking into account the follow-up parameters, body mass and BMI correlated with fasting insulin concentration (r = 0.63 and r = 0.68, respectively; *p* < 0.001), HOMA-β (r = 0.59 and r = 0.55, respectively; *p* < 0.05), HOMA-IR (r = 0.53 and r = 0.60, respectively; *p* < 0.05), and serum TG (r = 0.45 and r = 0.42, respectively; *p* < 0.05). A positive correlation between TG and HOMA-IR and fasting insulin concentration (both r = 0.41, *p* < 0.05) was also observed. All baseline miRNA expressions also correlated with patients’ fasting glucose and glucose concentration at 120′ during OGTT measured at visit 2. In the case of a correlation between fasting glucose and miR-298, the Spearman coefficient was r = 0.48; fasting glucose and miR-491-5p, r = 0.57; fasting glucose and miR-1307-3p, r = 0.44 (*p* < 0.01 for all). The Spearman correlations between glucose concentration at 120′ during OGTT and miR-298, miR-491-5p, and miR-1307-3p were r = 0.58, r = 0.65, and r = 0.41, respectively (*p* < 0.05 for all) (Figure 4B).

### 3.7. Receiver Operating Characteristic Curve Analysis

**Figure 5:**
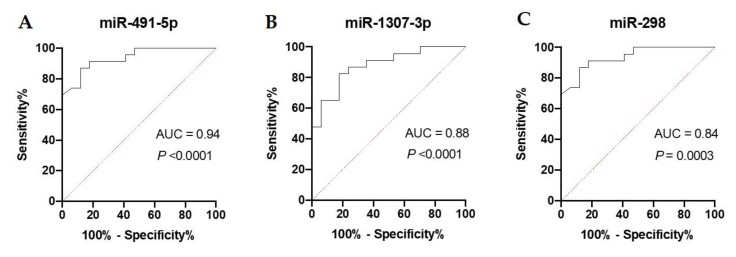
ROC analysis was constructed to evaluate the diagnostic values of the selected miRNAs as predictive biomarkers of type 2 diabetes: (**A**) miR-491-5p; (**B**) miR-1307-3p; (**C**) miR-298.

The diagnostic value of the candidate miRNAs as predictive T2DM biomarkers was evaluated by the AUC of the ROC curve. The AUC for all the DE miRNAs reached statistical significance compared to AUC = 0.5 (*p* < 0.001). The highest AUCs and, hence, possible clinical applicability, were observed for miR-491-5p, miR-1307-3p and miR-298, respectively (Figure 5A–C).

### 3.8. Logistic Regression Model

To investigate the eventual increase of diagnostic value by simultaneous consideration of multiple DE miRNAs, logistic regression models were developed using leave-one-out-cross-validation (LOOCV). Four models, based on various combinations of the three miRNAs with the highest diagnostic values, were prepared. Based on independent miRNAs belonging to different clusters—and based on hierarchical clustering—four logistic regression models were prepared. The parameters of the models and the common quality measures are summarized in Table 3. The highest AUC was obtained for a combination of miR-298 with miR-1307-3p and miR-491-5p (AUC = 95.7%); and this model had a higher diagnostic value compared to the highest AUC for miRNA used separately—miR-491-5p.

### 3.9. Data Validation

**Figure 6:**
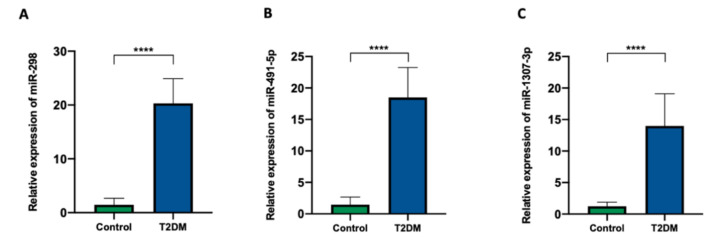
Relative mRNA expressions of the different miRNAs in the serum samples. (**A**) miR-298; (**B**) miR-491-5p; (**C**) miR-1307-3p. Each bar represents the mean ratio of gene expression and miR-103a-3p and miR-16-5p ± standard error of the mean (SEM). Asterisks indicate a significant difference compared to the control (**** *p* ≤ 0.0001).

To further verify the results of the NanoString analysis, the upregulated miRNAs from the miRNA profiling (miR-298, miR-1307-3p, and miR-491-5p) were then validated. For the validation experiments, candidate miRNAs were quantified using TaqMan probes. When compared with the expression profiles as established by NanoString, the qRT-PCR validation results demonstrate high similarity between the expression patterns of these miRNAs, determined using these two techniques (Figure 6A–C).

## 4. Discussion

It is important to acknowledge that in presented study, for dysglycemia diagnosis we used fasting and 2-h glucose criteria. Diagnostic criteria for diabetes have changed over the last several years. Traditionally, these criteria were based on thresholds of fasting plasma glucose concentration, plasma glucose concentration at 2-h of the oral glucose tolerance test (OGTT) and random plasma glucose concentration with the presence of classic symptoms of hyperglycemia. In the last decade, glycated hemoglobin (HbA1c) was added as an additional criterion of diabetes diagnosis. Moreover, in 2010, because of the standardization and widespread use of HbA1c, the American Diabetes Association (ADA) advocated for the use of glycated hemoglobin in the diagnosis of prediabetes (criterion: HbA1c = 5.7–6.4%). However, a limitation of HbA1c is that its accuracy is adversely affected by hemolysis, older age, non-white race, high dietary fat intake, alcohol consumption, cigarette smoking, liver disease, kidney disease and iron deficiency, independently of glycaemia [44]. Due to insufficient evidence and methodological issues of HbA1c concentration measurement, it was not adopted as one of diagnostic criteria of dysglycemia by the Diabetes Poland and not used in our study. We are aware that this might be a limitation of presented results.

T2DM is a complex metabolic disorder that involves multiple genes that affect diverse cellular signaling pathways. Commonly known biomarkers identify patients who already show metabolic alterations, such as hyperglycemia. Almost everyone who develops T2DM usually has prediabetes first, but not all patients with prediabetes end up with diabetes. Changes in lifestyle or the introduction of pharmacological intervention programs can significantly delay or even prevent T2DM development [45,46]. However, it has recently been shown that some patients may be at higher risk of T2DM onset that cannot be explained by lifestyle and metabolic factors [47]. It can be hypothesized that genetic factors play a role in the progression form prediabetes to diabetes. miRNAs, as important mediators of cell-to-cell communication and coordinators of many biological functions, could be useful markers and thus suitable to implement the monitoring and identifying of high-risk individuals for T2DM development before the metabolic imbalance sets in [48]. Thus, new potential miRNA biomarkers indicative of developing diabetes in the future are needed. Previous research has suggested that there are several serum miRNAs involved in the T2DM pathomechanism, including miR-126 [25,49,50,51], miR-375 [27], miR-15a [24], miR-223 [52], and miR-146a [53]. It has also been shown that circulating miR-122 levels are associated with the future development of the metabolic syndrome and T2DM in the general population [54]. miR-9, miR-28-3p, miR29a, miR-103, miR-30a-5p, and miR-150 plasma levels in combination with HbA1c could also identify individuals at risk [24]. Yang et al. suggested serum miR-23a as a biomarker for the early detection of T2DM and prediabetes with normal glucose tolerance [55].

Our study revealed the differential expression of three miRNAs in prediabetes patients who developed T2DM after five years in comparison to prediabetes patients who did not progress to T2DM. miR-491-5p, miR-1307-3p, and miR-298, were proven to predict well the T2DM onset years before its manifestation. ROC analysis was performed to assess the diagnostic value of the selected miRNAs as predictive biomarkers of T2DM. The highest AUC was observed for miR-491-5p. This raises the potential clinical utility of serum miRNA profiling and highlights the role of miRNAs as potential biomarkers for predicting individuals at risk of T2DM. The combined analysis showed that a logistic regression model consisting of miR-298, miR-1307-3p, and miR-491-5p can demonstrate higher diagnostic accuracy than miR-491-5p individually, which suggests that there is a need to introduce such a panel in predicting T2DM development in prediabetes patients, as miRNAs combination shows the highest diagnostic power.

There is little experimental evidence to link the studied miRNAs to T2DM; however, they have been previously reported as diagnostic or therapeutic targets. miR-298 has been shown to modulate neurite growth and beta-amyloid precursor protein-converting enzyme 1 involved in the pathogenesis of Alzheimer’s disease (AD) [56,57,58], which raises a question about the specific role of miR-298 in islet amyloid polypeptide (IAPP) aggregation in β-cell during T2DM development. Currently, growing evidence demonstrates a close relationship between T2DM and neurodegenerative diseases; therefore, it is hypothesized that mitochondrial dysfunction and oxidative stress might play a crucial role in T2DM development, such as in neurodegenerative diseases [59,60,61]. The amyloid cascade hypothesis exists as a pathogenic explanation of AD, where it is believed that cellular accumulation of IAPP triggers a cascade of destructive events, including mitochondrial dysfunction [62]. However, some evidence exists that indicates that mitochondrial dysfunction may be a primary causal event [63]. In vivo animal studies have shown that pancreatic islets in T2DM share much in common with the neuropathology in neurodegenerative diseases such as AD [64]. It has been shown that IAPP aggregation correlates with β-cell apoptosis, and low-grade islet inflammation contributes to the etiopathology of T2DM [65,66]. Studies have also reported that miR-491-5p serves as a tumor suppressor by targeting some cancer-related genes in gastric, cervical, and breast cancers [67,68,69,70]. Similarly, miR-1307-3p expression regulates cell proliferation in hepatocellular, colon, and breast cancers [71,72,73].

The IPA results suggested that nNOS signaling in neurons, amyloid processing, and hepatic cholestasis are mainly involved in the progression from prediabetes to T2DM. Prolonged hyperglycemia may lead to oxidative stress and low-grade inflammatory responses, including the neuroinflammatory response. Hypersecretion of amylin (amyloidogenic protein) and its accumulation in oligomerized form, similar to AD, has been observed in diabetic rats [74]. Deposits of aggregated IAPP are present in the pancreas of a great majority of T2DM patients, thus representing a histopathological hallmark of the disease [75,76]. Although IAPP has emerged as a novel player in T2DM pathology, the mechanisms of the intracellular accumulation of IAPP oligomers and IAPP-mediated toxicity in β-cells still remains unclear [77]. Another mechanism involved in T2DM progression is suggested to be nNOS signaling in neurons. nNOS constitutes the predominant source of NO in neurons and localizes to synaptic spines. Additionally, nNOS is also expressed in the endothelium of blood vessels within skeletal muscles, cardiac muscles, and smooth muscles, where NO controls blood flow and muscle contractility [78]. In vivo studies on retinal neurons in mice have demonstrated a role of increased nNOS activity in the early neuronal dysfunction of diabetic retinopathy [79]. One of the main pathways identified by IPA analysis is also hepatic cholestasis. Despite the fact that the relationship between T2DM and hepatic cholestasis has not been well discussed, the contribution of bile acids to the regulation of glycemic responses in T2DM has been demonstrated [80,81]. 

To identify key DE miRNA target genes, a PPI network was constructed with Cytoscape software and the cytoHubba plugin. *FBXO41*, *FBXO10*, *FBXW12*, *CDC26*, *TRIM41*, *KCTD7*, *ANAPC13*, *ASB13*, *UBE2C*, and *FZR1* were identified as hub genes. *FBXO41*, *FBXO10*, and *FBXW12* belong to the F-box family of proteins that share a common F-box domain. F-box proteins (FBPs) are the main functional components of the ubiquitin–proteasome system responsible for protein degradation [82]. Although all these genes have been identified thus far, only a few of them have been well characterized. *FBXO41* is involved in axon growth regulation and neuronal migration in the developing rat cerebellum [83]. The E3 ubiquitin ligase *FBXO10* regulates receptors for advanced glycation end product (RAGE) protein stability. It has been shown that the production of advanced glycation end products (AGEs) is upregulated in hyperglycemic conditions. AGEs and other RAGE ligands have been shown to play a crucial role in the genesis and maintenance of diabetic complications [84,85]. It has been demonstrated that FBPs are target genes of FoxO transcription factors that play a major role in diabetes-induced muscle atrophy [86,87,88]. The tripartite motif (TRIM) proteins are a group of ubiquitin E3 ligases that play critical roles in innate immune signaling, as well as in intrinsic immunity inhibiting viral infection [89]. *TRIM41* targets nucleoproteins for ubiquitination and protein degradation [90]. Little is known about the association between *ANAPC13* and T2DM; however, it has been suggested to be one of the key differentially expressed genes in the livers of T2DM patients [91]. *UBE2C* overexpression is involved in the mis-segregation of chromosomes and alters the cell cycle process, facilitating cell proliferation. Moreover, it has also been reported that *UBE2C* overexpression correlates with progression and poor prognosis in many tumors [92].

In addition, the miRNA target gene functional analysis indicated several biological pathways implicated in T2DM development. The GO analysis suggested that cell junction, transcriptional activator activity, and stress fiber assembly play an important role in the mechanism of T2DM development, which is consistent with the hub genes analysis. The top five diseases identified by IPA confirmed the contribution of the abovementioned pathways, not only to T2DM, but also to muscle autophagy. Currently, muscle autophagy is considered rather as a T2DM complication than as a prediabetes symptom [93]. The ubiquitin–proteasome, autophagy, and proteolytic pathways are involved in protein degradation in muscles, contributing to muscle atrophy [94]. A recent study by Sambashivaiah et al. showed a similarity in skeletal muscle mass, strength, and contractile quality between prediabetes and T2DM patients [95]. These results, which are coherent with our analysis, suggest that muscle atrophy develops years before T2DM development. However, the dysregulation of the muscle atrophy pathway in prediabetes patients needs detailed molecular study to fill in the data gaps. 

As T2DM development is gradual and lasts for years, sensitive parameters are needed for the screening of prediabetes patients. miRNAs have become useful markers for the diagnosis, prognosis, and therapeutic strategies of many conditions, such as endocrine cancers (e.g., breast and prostate cancers), lung cancer, melanoma, and cardiovascular and neuronal diseases [18,96,97,98,99,100]. There is a need to differentiate and separate miRNA analysis from blood, i.e., from all extracellular vesicles with free miRNAs and from the fluids where only free miRNA profiles can be evaluated. Depending on the material being tested, different miRNA fractions with distinct physiological functions are determined. The study performed by Chen et al. showed that serum miRNAs were more susceptible to changes in the ongoing case of diabetes than blood cell miRNAs [98]. To evaluate the miRNA profiles that predict the progression from prediabetes to T2DM, NanoString analysis in prediabetes patients was performed. The results indicated that the dysregulation of serum miRNA occurs years before the glycemia imbalance. The current study found that 18 miRNAs were differentially expressed in patients who later progressed to T2DM compared to those who did not. No association of the selected miRNAs’ expression with the baseline glycemic stage suggests their negligible role in glucose metabolism regulation. Serum miRNA profiling may be a useful tool for the non-invasive screening of prediabetic patients. RT-qPCR assay remains the most commonly used approach for miRNA expression profiling due to its sensitivity and specificity, and for these reasons, it was used for the NanoString data validation. The RT-qPCR analysis showed significantly increased expressions of miR-491-5p, miR-1307-3p, and miR-298 in the serum of patients who developed T2DM after five years versus non-T2DM patients, confirming the robustness of the nCounter platform.

## 5. Conclusions

Our results indicate that serum circulating miR-491-5p, miR-1307-3p, and miR-298 can be introduced as a diagnostic tool for the prediction of T2DM. These data should be viewed as preliminary; however, our study revealed candidate miRNAs deregulated years before T2DM development. Nonetheless, additional studies will be important for understanding the role of circulating miRNAs in the molecular mechanisms underlying T2DM and for identifying at-risk individuals.

## Figures and Tables

**Table 1:** Characteristics of the studied group (median and range).

	Control *N* = 18		T2DM *N* = 24			
Variable	Visit 1 ^a^	Visit 2 ^b^	Visit 1 ^a^	Visit 2 ^b^	*p-*Value Visit 1 ^c^	*p-*Value Visit 2 ^d^
Age [years]	54.57 (33.43–65.91)	56. 36 (37.36–70.96)	58.30 (28.88–65.12)	62.50 (41.16–69.20)	0.157	0.146
Female/Male	9/9		11/13			
BMI [kg/m^2^]	32.40 (25.51–46.05)	32.48 (23.66–47.05)	31.52 (22.85–47.22)	32.10 (26.71–49.35)	0.187	0.219
Weight [kg]	98.75 (68.8–145.9)	101.55 (64.00–144.10)	89.30 (60.20–162.10)	90.55 (65.80–176.30)	0.113	0.160
Fasting glucose 0 min [mg/dL]	106.50 (100.00–117.00)	108.00 (101.00–121.00)	107.00 (84.00–128.00)	129.00 (100.00–171.00)	0.876	0.0018
Glucose 120 min [mg/dL]	124.50 (68.00–182.00)	127.00 (72.00–190.00)	141.40 (69.00–186.00)	206 (160.00–229.00)	0.132	0.0001
Insulin [µU/mL]	13.49 (5.60–29.00)	15.00 (10.00–35.00)	12.00 (6.00–35.00)	16.00 (4.70–59.00)	0.618	0.88
HbA1c [%]	5.80 (4.10–6.40)	5.80 (5.10–6.40)	5.95 (4.90–6.60)	6.15 (5.30–7.70)	0.308	0.0057
LDL cholesterol [mg/dL]	123.10 (94.00–228.00)	105.00 (53.00–222.00)	118.50 (57.00–213.00)	95.50 (60.00–213.00	0.471	0.348
Total cholesterol [mg/dL]	196.00 (166.00–324.00)	181.00 (125.00–284.00)	191.00 (129.00–321.00)	174.00 (138.00–310.00)	0.458	0.723
HDL cholesterol [mg/dL]	49.50 (32.00–107.00)	49.70 (29.00–125.00)	51.70 (40.00–71.00)	53.00 (36.00–89.00)	0.517	0.319
Triglyceride [mg/dL]	132.00 (41.00–227.00)	107.00 (33.00–229.00)	112.50 (45.00–491.00)	124.50 (44.00–232.00)	0.131	0.875
HOMA-IR	3.40 (1.50–7.80)	4.30 (2.80–10.40)	2.95 (1.52–9.40)	5.20 (1.10–20.00)	0.783	0.479
HOMA-B	112.38 (43.00–275.00)	112.00 (71.00–216.00)	97.00 (42.23–349.00)	85.00 (19.00–277.00)	0.687	0.112

**^a^** visit 1 was performed from 2009–2012; **^b^** visit 2 was performed 5 years after the first visit; **^c^** p-value visit 1—difference between control and type 2 diabetes mellitus (T2DM) (based on the Mann–Whitney U test); **^d^** p-value visit 2—difference between control and T2DM (based on the Mann–Whitney U test); BMI, body mass index; HbA1c, glycated hemoglobin A1c; LDL cholesterol, high-density lipoprotein cholesterol; HDL cholesterol, high-density lipoprotein cholesterol; HOMA-IR, homeostasis model assessment for insulin resistance; HOMA-B, homeostasis model assessment for beta (β) cell function.

**Table 2:** The list of serum miRNAs with significantly different expressions at the baseline between groups (FC > 1.5; FDR  ≤  0.05).

miRNA	FC	FDR
**miR-298**	1.95	0.05
**miR-491-5p**	1.95	0.01
**miR-1307-3p**	1.85	0.02

FC, fold change; FDR, false discovery rate.

**Table 3:** Summary of the basic parameters and common quality measures of the models.

	AUC (CI)	Cut-Off Point	Specificity	Sensitivity	Accuracy	TP/TN/FP/FN	Intercept (a_0_)	Coefficients
x1 = miR-298	95.70%	1.5	100%	91.30%	0.95	17/2/0/21	–649.78	a1 = 31.25
x2 = miR-1307-3p								a2 = 27.99
x3 = miR-491-5p								a3 = 81.50
x1 = miR-298	82.50%	1.5	82.35%	82.60%	0.83	14/4/3/19	−5.99	a1 = 0.64
x2 = miR-1307-3p								a2 = 0.70
x1 = miR-298	92.70%	1.5	94.20%	91.30%	0.93	16/2/1/21	−16.81	a1 = 1.32
x2 = miR-491-5p								a2 = 2.53
x1 = miR-1307-3p	87.60%	1.5	88.23%	86.96%	0.86	15/3/2/20	−23.09	a1 = 1.63
x2 = miR-491-5p								a2 = 3.06

AUC, area under the curve (AUC) of the receiver operating characteristic (ROC) curves; CI, confidence interval; FN, false negative; FP, false positive; TN, true negative; TP, true positive.
